# MiR-194 functions as a tumor suppressor in laryngeal squamous cell carcinoma by targeting Wee1

**DOI:** 10.1186/s13045-017-0402-6

**Published:** 2017-01-25

**Authors:** Pei Li, Yang Yang, Hui Liu, An-Kui Yang, Jin-Ming Di, Guang-Mou Tan, Hai-Feng Wang, Jian-Ge Qiu, Wen-Ji Zhang, Qi-Wei Jiang, Di-Wei Zheng, Yao Chen, Meng-Ning Wei, Jia-Rong Huang, Kun Wang, Zhi Shi, Jin Ye

**Affiliations:** 1 Department of Otolaryngology-Head and Neck Surgery, The Third Affiliated Hospital, Sun Yat-sen University, Guangzhou, Guangdong China; 2 Department of Cell Biology & Institute of Biomedicine, National Engineering Research Center of Genetic Medicine, Guangdong Provincial Key Laboratory of Bioengineering Medicine, College of Life Science and Technology, Jinan University, Guangzhou, Guangdong China; 3 Division of Pulmonary and Critical Care, Department of Internal Medicine, The Third Affiliated Hospital, Sun Yat-sen University, Guangzhou, Guangdong China; 4 Department of Head and Neck, State Key Laboratory of Oncology in South China, Collaborative Innovation Center for Cancer Medicine, Sun Yat-sen University Cancer Center, Guangzhou, Guangdong China; 5 Department of Urology, The Third Affiliated Hospital, Sun Yat-sen University, Guangzhou, Guangdong China; 6 Department of Head & Neck Surgery, Affiliated Cancer Hospital & Institute of Guangzhou Medical University, Guangzhou, China

**Keywords:** Laryngeal squamous cell carcinoma, miR-194, Wee1

## Abstract

The emerging roles of microRNAs (miRs) have been deeply investigated in cancer. However, the role of miR-194 in human laryngeal squamous cell carcinoma (LSCC) is still unclear. Here, we have demonstrated that miR-194 is significantly downregulated in LSCC tissues and cells, and overexpression of miR-194 inhibits the proliferation, migration, invasion, and drug resistance in LSCC cells. Moreover, Wee1 is identified as a novel direct target of miR-194. Ectopic expression of Wee1 at least in part overcomes the suppressive impacts of miR-194 on the malignant phenotypes of LSCC. Overall, our study provides new sights into the role of miR-194/Wee1 axis in LSCC and suggests a novel miR-194/Wee1-based clinical application for LSCC patients.

**Electronic supplementary material:**

The online version of this article (doi:10.1186/s13045-017-0402-6) contains supplementary material, which is available to authorized users.

## Letter to the editor

Laryngeal cancer is the fourteenth most prevalent type of malignancy worldwide in the male compared to its relative rare in the female. Laryngeal squamous cell carcinoma (LSCC) accounts for approximately 90% of all malignant tumors of the larynx [1]. Deregulated microRNAs (miRs) are frequently demonstrated as biomarker or therapeutic target in LSCC tissues and cells, which may function as tumor suppressor or oncogene to regulate the malignancy of cancer [2]. As one of frequently deregulated miRs in cancer, the expression and role of miR-194 in LSCC are still unknown [3, 4].

**Fig. 1:**
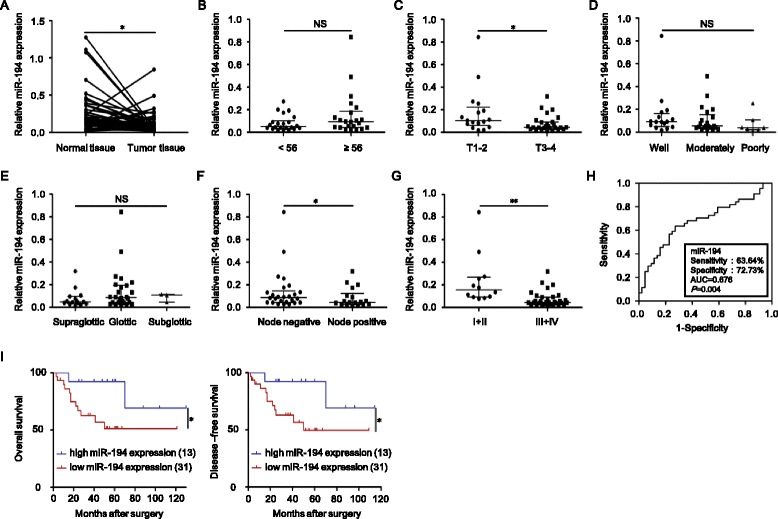
Downregulation of miR-194 in LSCC is correlated with T stages, lymph node metastasis, clinical stages, recurrence, and poor prognosis. **a** RT-qPCR analysis of the relative miR-194 expression in 44 pairs of LSCC tissues and adjacent normal tissues with Student’s *t* test. The relative miR-194 expression in two groups of LSCC tissues classified by age (**b**), T stage (**c**), lymph node metastasis (**f**) and clinical stage (**g**) were analyzed with Mann-Whitney *U* test. The relative miR-194 expression in three groups of LSCC tissues classified by differentiation (**d**) and primary location (**e**) were analyzed with Kruskal–Wallis test. **h** ROC curve analysis of the discrimination between LSCC tissues and adjacent normal tissues by miR-194. **i** Kaplan–Meier analysis of overall survival and disease-free survival curves for LSCC patients with high and low expression of miR-194. Data are presented as mean ± SD or median with the interquartile range. *P < 0.05; **P < 0.01; *NS* no statistical significance

**Fig. 2:**
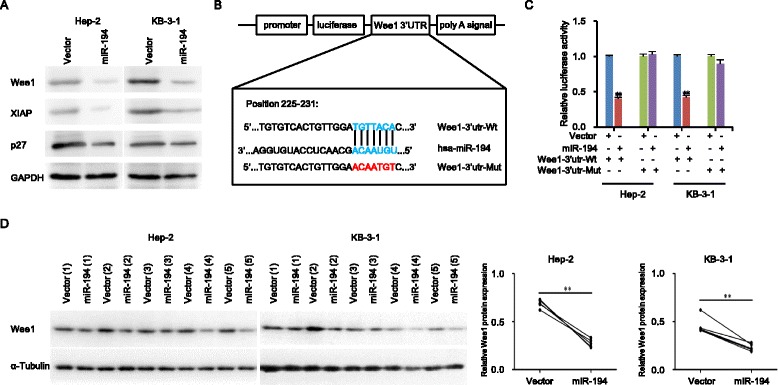
MiR-194 directly targets Wee1. **a** Western blot analysis of Wee1, XIAP, and p27 protein expressions in the indicated cells. GAPDH is the loading control. **b** A schematic diagram of the reporter constructs showed the wild type (Wt) and mutant (Mut) sequences of the miR-194 binding sites within human Wee1 3’-UTR. **c** Luciferase activity of reporters with Wee1 Wt or Mut 3’-UTR in the indicated cells. **d** Western blot analysis and quantification of Wee1 protein expression in the tumors of the indicated cells. A-Tubulin was the loading control. Data are presented as mean ± SD. Student’s *t* test was used for statistical analysis. **P < 0.01

In the current study, we found that the expression level of miR-194 is significantly lower not only in two LSCC cell lines Hep-2 and KB-3-1 compared with normal human bronchial epithelial cell line 16HBE but also in clinical LSCC tissues compared with adjacent normal tissues and correlated with T stage, lymph node metastasis, and clinical stage, but not with age, tumor grades, and tumor primary locations (Fig. 1b–g, Additional file 1: Figure S1a and Table S1). The miR-194 levels could be a significant parameter to distinguish LSCC and adjacent normal tissues with an area under the ROC curve (AUC) of 0.676 (sensitivity = 63.64%, specificity = 72.72%; *P* = 0.004) (Fig. 1h). Kaplan–Meier analysis indicated that high miR-194 expression predicts a favorable outcome for LSCC patients (Fig. 1i). Functional assays showed that enforced expression of miR-194 inhibits the growth, migration, invasion, and drug resistance of Hep-2 and KB-3-1 cells in vitro (Additional file 1: Figure S2a–g). The data in the subcutaneous tumor model in nude mice revealed that overexpression of miR-194 significantly inhibited the growth of Hep-2 and KB-3-1 xenografts by the numbers of Ki67^+^ proliferating cells and CD31^+^ microvessels (Additional file 1: Figure S3a–d). We used three computational algorithms, including miRanda, PITA, and TargetScan in combination to identify the novel potential targets of miR-194. All three algorithms predicted Wee1 as a target gene of miR-194, which is a protein kinase to regulate cell cycle through inhibiting CDK1 by phosphorylation on its two different residues Thr14 and Tyr15 [5]. Western blot analysis showed that ectopic expression of miR-194 in Hep-2 and KB-3-1 cells obviously downregulated the protein levels of Wee1 as well as the known targets XIAP and p27 [3, 6] (Fig. 2a). The predicted interaction between miR-194 and the target sites within the 3’-untranslated regions (3’UTR) of Wee1 was shown in Fig. 2b. We then performed luciferase assay to examine whether there is a direct interaction between miR-194 and Wee1. The wild type or mutant 3’UTR region of Wee1 were cloned into downstream of the firefly luciferase gene to generate the luciferase reporter vectors. As illustrated in Fig. 2c, ectopic expression of miR-194 significantly decreased the luciferase activity of wild type 3’UTR, but not mutant 3’UTR in Hep-2 and KB-3-1 cells, suggesting that the putative miRNA binding sites of Wee1 are responsible for this miRNA-mRNA interaction (Additional file 1: Figure S1b). Furthermore, analysis of proteins extracted from Hep-2 and KB-3-1 subcutaneous tumors in mice exhibited that the protein levels of Wee1 in miR-194-transduced tumors were remarkably lower than those in vector control (Fig. 2d). In addition, ectopic expression of Wee1 partially reverses the suppressive effects of miR-194 on LSCC cells (Additional file 1: Figure S4a–g).

In summary, our data reveal a potential suppressive role of miR-194 in LSCC by targeting Wee1 in vitro and in vivo. The clinical results indicate that miR-194 can be the potential diagnostic and prognostic biomarkers for LSCC. Our study provides new sights into the role of miR-194/Wee1 axis in LSCC and suggests a novel miR-194/Wee1-based clinical application for LSCC patients.

## Additional file

Additional file 1: Supplemental data. (PDF 1209 kb)

## Abbreviations

LSCC: Laryngeal squamous cell carcinoma
UTRs: 3’-untranslated regions
