# Supplementary material for: MiR-194 functions as a tumor suppressor in laryngeal squamous cell carcinoma by targeting Wee1
Source: J Hematol Oncol. 2017 Jan 25;10:32. doi: 10.1186/s13045-017-0402-6 (PMC5264457; doi:10.1186/s13045-017-0402-6)

Methods

Patients and specimens

A total 44 pairs of LSCC and corresponding adjacent normal tissues were obtained from patients who underwent partial or total laryngectomy without neoadjuvant radical or chemical therapy before and after surgery at the Department of Head and Neck, Sun Yat-sen University Cancer Center, Guangzhou, China from July 2008 to June 2015. The International Union Against Cancer (UICC) 2002 norms for staging laryngeal carcinoma (clinical, endoscopic, and imaging) is strictly followed. Signed informed consents were obtained from all patients. The study was approved by the ethics committee of Sun Yat-sen University Cancer Center.

Cell culture and reagents

The human LSCC cell lines Hep-2, KB-3-1, normal bronchial epithelium cell line 16HBE and embryonic kidney cell line HEK293T were cultured in Dulbecco's modified Eagle's medium (DMEM) with 10% fetal bovine serum (FBS), penicillin (100 U/ml) and streptomycin (100 ng/ml) at 37℃ with 5% CO_2 in a humidified incubator. Puromycin, doxorubicin (DOX) and cisplatin (DDP) were purchased from ApexBio and Qilu Pharmaceutical, respectively. Anti-Wee1 (SC-5285) antibody was from Santa Cruz Biotechnology. Anti-XIAP (2808) and anti-p27 (3698) antibodies were from Cell Signaling Technologies. Anti-Ki-67 (RLT2467) and anti-CD31 (RLT0752) antibodies were from Ruiying Biological. Anti-GAPDH (KM9002) antibody was from Tianjin Sungene Biotech. Anti-α-Tubulin (4777) antibody was from Ruiying Biological.

RNA extraction and real-time quantitative PCR (RT-qPCR)

Total RNA was extracted from cells and tissues using HiPure Total RNA Mini Kit (Magen) according to the manufacturer's protocol. Reverse transcription was performed with HiFi-script cDNA kit (Cwbio) according to the manufacturer's instruction. The BestarTM Real time PCR Master Mix was used for RT-qPCR by SYER Green Method. All reactions were performed on an ABI 7900HT instrument (Applied Biosystems). All reactions were run in triplicate and all experiments were carried at least three independent times. The results of RT-qPCR were normalized to U6 using the $2^{-\Delta\Delta Ct}$ method. The primers were ordered from Sangon Biotech. Primer sequences are shown in Supplementary Table 2.

MTT assay

Cells were seeded into a 96-well plate at a density of 0.5–1 $\times 10^4$ cells/well and treated with various concentrations of agents. After 3 days, 3-(4, 5-dimethylthiazolyl-2)-2, 5-diphenyltetrazolium bromide (MTT) was added to each well at a final concentration of 0.5 mg/ml. After incubation for 4 hours, the medium and MTT solution were removed from each well, and formazan crystals were dissolved in 100 μl of DMSO. Absorbance was measured at 570 nm by Multiscan Spectrum (Thermofisher).

Western blot analysis

Cells were harvested and washed twice with cold PBS, then resuspended and lysed in RIPA buffer (1% NP-40, 0.5% sodium deoxycholate, 0.1% SDS, 10 ng/ml PMSF, 0.03% aprotinin, 1μM sodium orthovanadate) at 4°C for 30 minutes. Lysates were centrifuged for 10 minutes at 14,000 × g and supernatants were stored at -80 ℃ as whole cell extracts. Proteins were separated on 12% SDS-PAGE gels and transferred to

polyvinylidene difluoride membranes. Membranes were blocked with 5% BSA and incubated with the indicated primary antibodies. Corresponding horseradish peroxidase-conjugated secondary antibodies were used against each primary antibody. GAPDH or α-Tubulin was used as a loading control. Signals were detected using the ChemiDoc XRS chemiluminescent gel imaging system (Bio-RAD).

Wound healing assay

Briefly, cells (5×10^5/well) were seeded into six-well dishes. Till the cells reached 80–90% confluence, the cell monolayer was wounded using a sterilized 10 μl pipette tip and washed with PBS two times. Cells were allowed to migrate for 24 hours and 48 hours in serum-free medium, and the wounds were observed and captured. The gap lengths were measured from the photomicrographs.

Transwell assay

Cells were plated in the upper compartment of a modified Boyden chamber (Corning) containing matrigel-coated polycarbonate membrane filter (6.5 mm diameter, 8 μm pore size), and the lower chamber contained medium with 10% FBS, and allowed to migrate for 24 hours at 37 ℃ in 5% CO_2. Cells on the upper surface of the membrane were wiped off, and cells invaded to the bottom surface were photographed and counted.

Sphere formation assay

Cells were trypsinized, suspended in medium containing 0.3% agar and 10% FBS and seeded at a density of 5×10^2 cells/well in a 12-well plate. The agar–cell mixture was plated onto a bottom layer with 0.5% agar. Then treated cells were incubated in a humidified incubator and fresh medium was added every 3 days. Two weeks later,

colonies were analyzed microscopically.

Cell cycle assay

Cells were digested, harvested and then washed twice with cold phosphate-buffered saline (PBS), then permeabilized with 70% cold ethanol for 2 hours at 4℃. After washing twice in PBS, cells were resuspended with 0.5 ml PBS containing PI (50 μg/ml), 0.1% Triton X-100, 0.1% sodium citrate, and DNase-free RNase (100 μg/ml), and assessed by FCM after incubation at room temperature in the dark for 15 minutes. Fluorescence was measured at an excitation wavelength of 480 nm through a FL-2filter (585 nm). Data were analyzed using ModFit LT 3.0 software.

Plasmid construction and lentivirus production

The synthesized precursor hsa-miR-194 (Supplementary Table 1) was cloned into lentiviral vector pLKO.1-GFP to generate the hsa-miR-194 lentivirus construct. The Wee1 cDNA was cloned into lentiviral vector LV5. Lentivirus was packaged in HEK293T cells and collected from the medium supernatant. Stable cell lines were established by infecting lentivirus into Hep-2 and KB-3-1 cells, followed by puromycin selection.

Nude mice tumorigenesis assay

Balb/c nude mice were obtained from the Shanghai SLAC Laboratory Animal Co and maintained with sterilized food and water. Five female nude mice with 5 weeks old and 16-18 g weight were used for each group. Hep-2 cells, as well as KB-3-1 cells, infected with control or has-miR-194 lentivirus, respectively, were suspended 4×10^6 in 100 μl of DMEM for each mouse and were injected subcutaneously under the shoulder of the

female nude mice, five mice per group. The body weights of the animals and the two perpendicular diameters (a and b) of tumor were recorded every five days. The tumor volume (V) was calculated as:

$$V = \frac{\pi}{6}\left(\frac{a+b}{2}\right)^3$$

The mice were anaesthetized after experiment,, and tumors were removed, weighed, and sectioned, followed by qRT-PCR analysis and IHC analysis. All experimental procedures were approved by the Institutional Animal Care and Use Committee of Sun Yat-Sen University.

Luciferase reporter assay

The pmirGLO, pmirGLO-Wee1 3'-UTR-wt or pmirGLO-Wee1 3'-UTR-mut (Promega) vectors were cotransfected into Hep-2 and KB-3-1 cells respectively with the hsa-miR-194 vector or empty vector. Cell lysates were collected at 24 hours posttransfection. Luciferase and renilla signals were measured using the Dual Luciferase Reporter Assay Kit (Promega) according to a protocol provided by the manufacturer.

Phalloidin staining assay

Cells were seeded on glass cover slips for 24 hours and then fixed in 4% paraformaldehyde for 20 minutes and permeabilized with 0.1% Triton X-100 for 15 minutes at room temperature. The coverslips were incubated in the dark with 100 nM rhodamine-phalloidin at room temperature for 30 minutes. Nuclei were counterstained with 100 nM DAPI. The coverslips were rinsed in PBS and inverted on a drop of anti-fade mounting media on a glass slide. Then, these slides were sealed with neutral balsam and viewed under the confocal microscope.

Statistical analysis

All statistical analyses were performed using the SPSS 20.0 statistical software package. Comparisons between two groups were performed using Student's t-test or Mann–Whitney U-test, and comparisons among three groups were performed using one-way ANOVA or Kruskal-Wallis test. Kaplan–Meier method and the log-rank test were used to compare the survival of patients. ROC curve analyses were used to evaluate the prognostic ability. The difference in tumor volume between the two groups of mice was determined by repeated-measures analysis of variance. Data were presented as mean ± SD or median with the interquartile range. $P<0.05$ was considered statistically significant.

Supplementary Table 1. Relationship between miR-194 expression level and clinicopathologic parameters in LSCC.

Characteristics (n)	miR-194 level[a]	p[b]
Age		0.1131
<56 (22)	0.0807 ± 0.0704	
≥56 (22)	0.1534 ± 0.1917	
T stage		0.022
T1-2 (17)	0.1794 ± 0.2074	
T3-4 (27)	0.0777 ± 0.0728	
Differentiation		0.5053
Well (16)	0.1479 ± 0.1970	
Moderately (21)	0.1082 ± 0.1179	
Poorly(7)	0.0728 ± 0.0858	
Primary location		0.2689
Supraglottic（15）	0.0757 ± 0.0788	
Glottic（26）	0.1441 ± 0.1785	
Subglottic（3）	0.0888 ± 0.0379	
Lymph node metastasis		0.0339
Negative (26)	0.1400 ± 0.1757	
Positive (18)	0.0839 ± 0.0869	
Clinical stage		0.0002
I+II（12）	0.2349 ± 0.2251	
III+IV（32）	0.0728 ± 0.0688	

[a]Scores determined by RT-qPCR in mean ± SD. [b]Mann-Whitney U-test (for 2 groups) or Kruskal-Wallis test (for > 2 groups).

Supplementary Table 2. Primers for reverse transcription and RT-qPCR.

Primers	Sequences (From 5' to 3')
U6-F	GCGCGTCGTGAAGCGTTC
U6-R	GTGCAGGGTCCGAGGT
U6-RT	GTCGTATCCAGTGCAGGGTCCGAGGTATTCGCACTGGATACGACAAAATA
hsa-miR-194-F	CACGCATGTAACAGCAAC
hsa-miR-194-R	CCAGTGCAGGGTCCGAGGTA
hsa-miR-194-RT	GTCGTATCCAGTGCAGGGTCCGAGGTATTCGCACTGGATACGACTCCACA
precursor hsa-miR-194-F	CCGGATGGTGTTATCAAGTGTAACAGCAACTCCATGTGGACTGTGTACCAATTTCCAGTGGAGATGCTGTTACTTTTGATGGTTACCAATTTTTG
precursor hsa-miR-194-R	AATTCAAAAATTGGTAACCATCAAAAGTAACAGCATCTCCACTGGAAATTGGTACACAGTCCACATGGAGTTGCTGTTACACTTGATAACACCAT
Wee1-F	CGATCGGGCCTGTAATTTGG
Wee1-R	TTGTCCAGAAACAGGCCTCA
β-actin-F	TCTGGCACCACACCTTCTAC
β-actin-R	CAGCTTCTCCTTAATGTCAC

Figure legends

Supplementary Figure 1. MiR-194 is significantly downregualted in LSCC cells. (**A**) RT-qPCR analysis of the relative miR-194 expression in the human LSCC cell lines Hep-2, KB-3-1 and normal bronchial epithelium cell line 16HBE. (**B**) Predicted precursor structure of miR-194. The Wee1 binding sites are highlighted in blue on the predicted hairpin precursor.

Supplementary Fig. 2 MiR-194 suppresses the growth, migration, invasion and drug-resistance of LSCC cells in vitro. (**A**) RT-qPCR analysis of the relative miR-194 expression in Hep-2 and KB-3-1 cells expressed vector control and miR-194. (**B**) Cell proliferation of the indicated cells as determined with MTT assay. (**C**) Cell survival of the indicated cells treated with DDP and DOX as determined with MTT assay. (**D**) Representative images and quantification of the indicated cells migration as determined with wound healing assay. (**E**) Representative images and quantification of the indicated cells invasion as determined with Transwell assay. (**F**) Representative images of the indicated cells cytoskeleton as determined with phalloidin staining. (**G**) Representative images and quantification of the indicated cells sphere as determined with sphere formation assay. (**H**) Representative images and quantification of the indicated cells cycle distribution as determined with flow cytometry. Data are presented as mean ± SD of three independent experiments. Student's t-test was used for statistical analysis. *P<0.05; **P<0.01.

Supplementary Fig. 3 MiR-194 inhibits the growth of LSCC cells *in vivo*. (**A**) The subcutaneous tumors of the indicated cells. The tumor volume and weight were determined by repeated-measures analysis of variance and Student's t-test respectively.

(**B**) RT-qPCR analysis of the relative miR-194 expression in the subcutaneous tumors of the indicated cells. Representative images and quantification of Ki-67^+ cells (**C**) and $CD31^+$ microvessels (**D**) in the the indicated tumors as determined with IHC assay and Student's t-test. Data are presented as mean ± SD. **P*<0.05; ***P*<0.01.

Supplementary Fig. 4 Wee1 partially reverses the suppressive effects of miR-194 on the growth, migration, invasion and drug-resistance of LSCC cells. (**A**) Western blot analysis of Wee1 protein expressions in the indicated cells. GAPDH is the loading control. (**B**) Cell proliferation of the indicated cells as determined with MTT assay. (**C**) Cell survival of the indicated cells treated with DDP and DOX as determined with MTT assay. (**D**) Representative images and quantification of the indicated cells migration as determined with wound healing assay. (**E**) Representative images and quantification of the indicated cells invasion as determined with Transwell assay. (**F**) Representative images and quantification of the indicated cells sphere as determined with sphere formation assay. (**G**) Representative images and quantification of the indicated cells cycle distribution as determined with flow cytometry. Data are presented as mean ± SD of three independent experiments. Student's t-test was used for statistical analysis. $^*P<0.05$; $^{**}P<0.01$.

Supplementary Figure 1

A

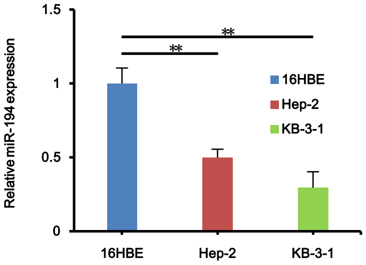

B

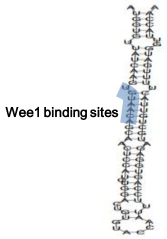

Precursor-hsa-miR–194

Supplementary Figure 2

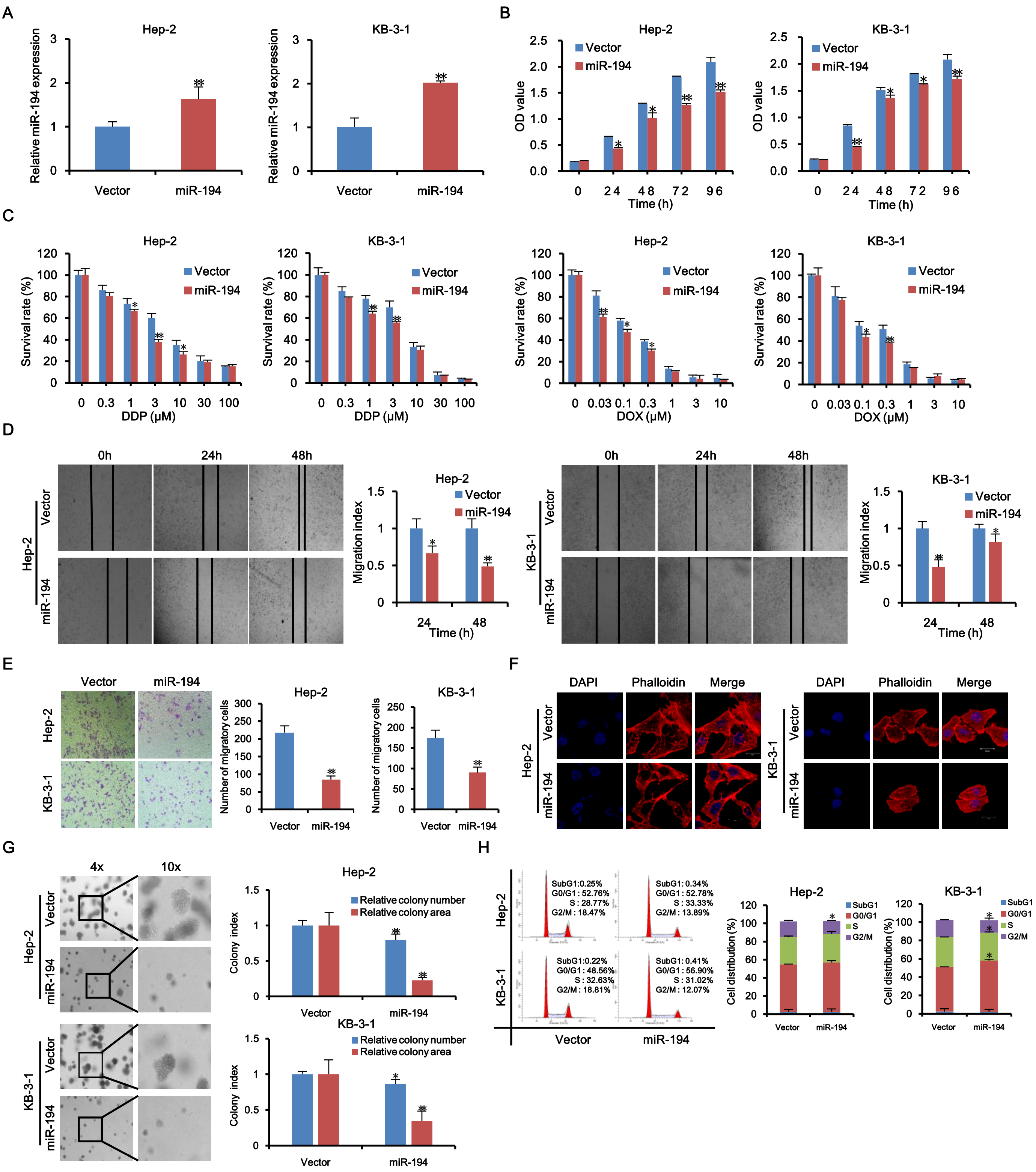

Supplementary Figure 3

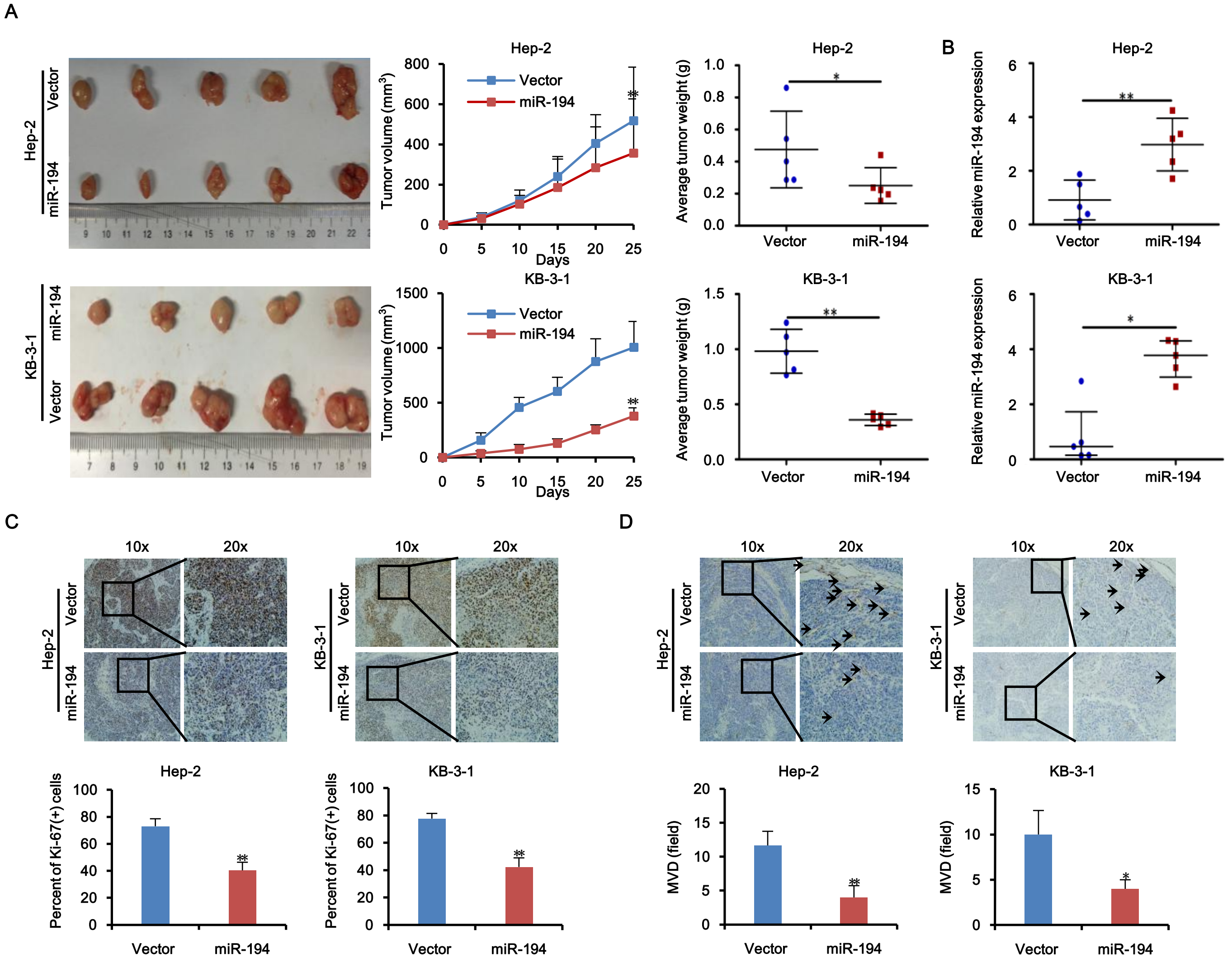

Supplementary Figure 4

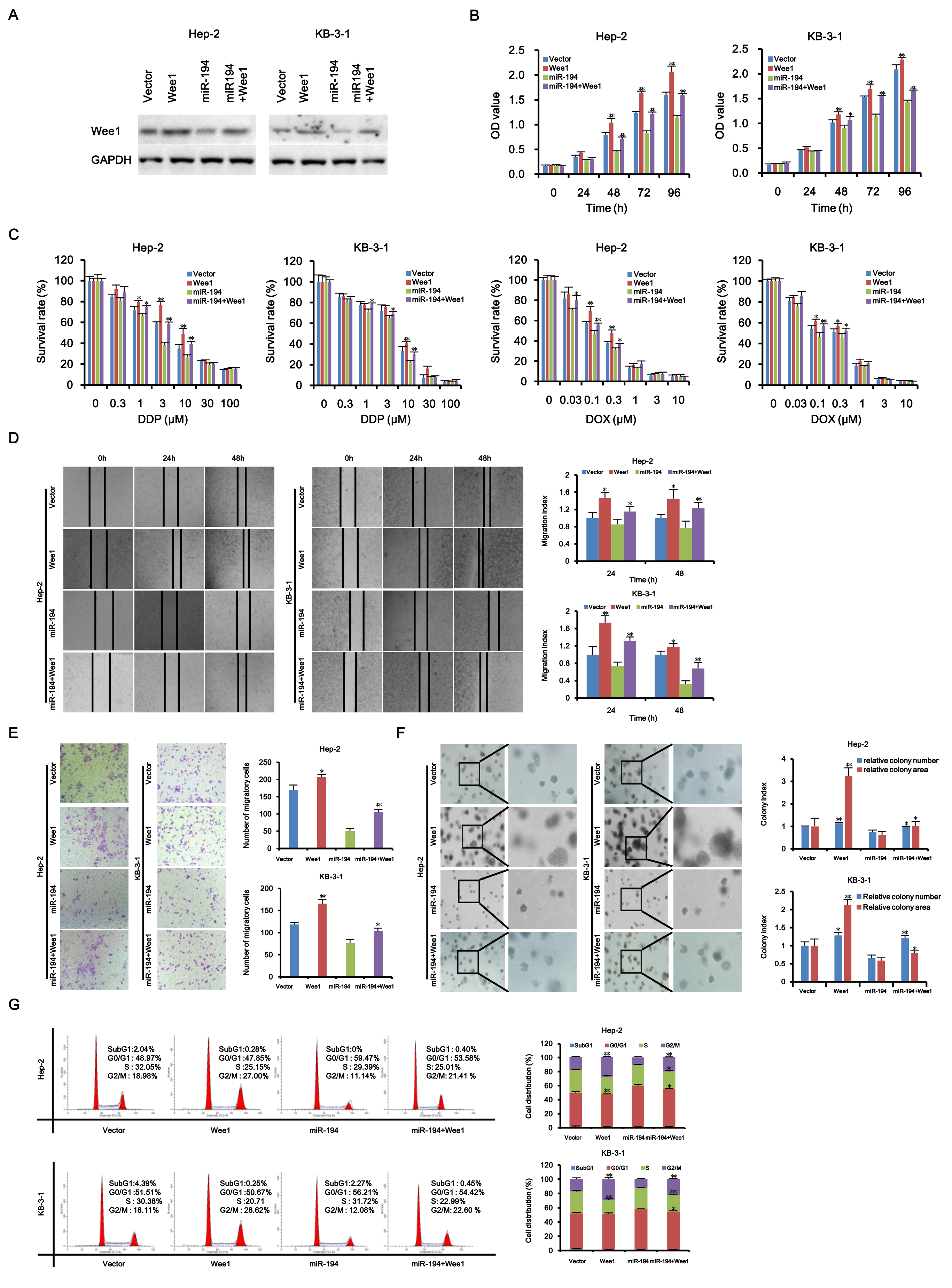

Supplement: Additional file 1: — Supplemental data. (PDF 1209 kb) [file 13045_2017_402_MOESM1_ESM.pdf]
